# Precision and Limits of Detection for Selected Commercially Available, Low-Cost Carbon Dioxide and Methane Gas Sensors

**DOI:** 10.3390/s19143157

**Published:** 2019-07-18

**Authors:** Wesley T. Honeycutt, M. Tyler Ley, Nicholas F. Materer

**Affiliations:** 1 Department of Chemistry, Oklahoma State University, 107 Physical Sciences, Stillwater, OK 74078, USA; 2 Department of Civil Engineering, Oklahoma State University, 319C Engineering South, Stillwater, OK 74078, USA

**Keywords:** sensor systems and applications, carbon dioxide, methane

## Abstract

The performance of a sensor platform for environmental or industrial monitoring is sensitive to the cost and performance of the individual sensor elements. Thus, the detection limits, accuracy, and precision of commercially available, low-cost carbon dioxide and methane gas concentration sensors were evaluated by precise measurements at known gas concentrations. Sensors were selected based on market availability, cost, power consumption, detection range, and accuracy. A specially constructed gas mixing chamber, coupled to a precision bench-top analyzer, was used to characterize each sensor during a controlled exposure to known gas concentrations. For environmental monitoring, the selected carbon dioxide sensors were characterized around 400 ppm. For methane, the sensor response was first monitored at 0 ppm, close to the typical environmental background. The selected sensors were then evaluated at gas concentrations of several thousand ppm. The determined detection limits accuracy, and precision provides a set of matrices that can be used to evaluate and select sensors for integration into a sensor platform for specific applications.

## 1. Introduction

Monitoring the concentrations of carbon dioxide (CO_2_) and methane (CH_4_), two important environmental gases, is necessary to understand their impact on the environment. Additionally, monitoring is critical to ensure worker safety by early identification of potential leaks. An example is monitoring CO_2_ and CH_4_ around carbon sequestration operations and oil fields. In such a scenario, collection of multiple samples at several locations in a given area is critical to obtain a reliable understanding of gas emission from the site (see Keith et al. [1] for aspects of effective sampling). Thus, cost becomes an important factor in the evaluations. For monitoring applications, available sensors include, but are not limited to, those used in HVAC air handlers [2,3], chemical processing units [4], oil well monitoring devices [5,6], and environmental monitoring [7,8,9,10,11]. In designing a monitoring platform, one must also consider the limit of detection, precision, accuracy, reliability, and power consumption for each sensor integrated within the unit. Given the large number of commercially available sensors and papers discussing their use in various applications, there is limited literature that directly compares sensors from different manufacturers. Although information can be obtained by the manufacturer of each sensor, in many cases the provided information is incomplete. For example, many sensors quote the resolution (in the case of a digital output) but do not report the limit of detection determined by the fluctuation of the baseline. In addition, manufacturers may have different methodologies and test their sensors under different conditions.

To address this issue, an array of commercially available, low-cost sensors for CO_2_ and CH_4_ were evaluated using the same methodology at concentrations typical to those required for environmental monitoring, and monitoring around carbon sequestration operations and oil fields. The sensors were evaluated at environmental concentrations of CO_2_ (around 400 ppm [12,13]) and CH_4_ (under 2 ppm [14,15,16]), and at concentrations of several thousand ppm, which simulates a potential leak. By evaluating these sensors at environmental concentrations and at concentrations simulating a potential leak, the results are transferable to a wide range of environmental and industrial monitoring applications. In this study, all testing was performed using controlled mixtures of pure gases. This way, the detection limits, accuracy, and precision of these sensors could be determined by precise measurements at known gas concentrations under identical and reproducible conditions. As this study focused on the performance of sensors to the analyte gas, sensitivity toward the specific target and other potential interference were not explored.

## 2. Methods

### 2.1. Sensor Selection

Selections were made from commercially available sensors. Potential sensors were categorized by detection method. Principal among these methods are optical absorption, chemiresistive (based on the resistance changes of a material due to chemical reaction with an analyte [17]), and electrochemical. Since studies have cited concerns with electrochemical gas concentration sensors, such as a short lifetime and lack of robustness [18], only optical and chemiresistive sensors were selected for evaluation in this study. Additional details on the sensing technology or detection method can be found in the Supplementary Materials. Briefly, NDIR detection is generally used for CO_2_ due to its relatively large molar absorption coefficient, allowing for short path lengths to be used in devices. Use of NDIR detectors for CH_4_ is limited in practical applications due to the lower absorption coefficient and overlapping symmetric C-H stretches. Thus, chemiresistive sensors are popular for the detection of CH_4_ and typically use a thin oxide film [18], on which the analyte adsorbs and reacts with surface oxygen species resulting in a change in the electrical conductivity [19,20,21]. Since the sensor depends on chemical reactions involving both atmospheric oxygen and CH_4_ for proper functioning, chemiresistive sensors are dependent on atmospheric properties such as relative humidity and temperature in addition to film preparation. Both optical and chemiresistive CH_4_ sensors can respond to a range of hydrocarbon gases. For optical sensors, overlapping stretches makes CH_4_ difficult to distinguish from other common aliphatic gases such as ethane and propane [22]. For chemiresistive sensors, similar reactivity results in sensitivity to a range of hydrocarbon gases [23].

With two exceptions, the sensors selected were all commercially available in large volumes (at least 1000 units) at low-cost (defined here as less than $100 per unit in bulk) Additional information on the selection rationale can be found in the Supplementary Materials. Selected sensors were expected to have sensitivity at the environmental concentrations of CO_2_ and CH_4_. For CO_2_, this level is around 400 ppm [12,13]. For CH_4_, the baseline atmospheric concentration is under 2 ppm [14,15,16]. In total, six CO_2_ sensors were selected for testing, the K-30 SE-0018, COZIR AMB GC-020, Gascard CO${}_{2}$, MSH-P/CO_2_/NC/5/V/P/F, MSH-DP/HC/CO_2_/NC/P/F, and Telaire T6615. Given the prevalence of optical sensors for CO_2_, no chemiresistive sensor was selected for this analyte. For CH_4_, seven sensors were evaluated, the MQ-4, Gascard CH${}_{4}$, MSH-P/HC/NC/5/V/P/F, MSH-DP/HC/CO_2_/NC/P/F, TGS-2600, TGS-2610, and TGS-2611. The relevant properties, obtained from the manufacturer documentation, for the CO_2_ (Table S1) and CH_4_ sensors (Table S2) are summarized in the Supplementary Materials. The selected sensors meet the requirements for use in portable low-power monitoring devices [24].

The K-30, COZIR, Dynament, and Telaire sensors are all NDIR CO_2_ sensors. The Dynament hydrocarbon sensor (MSH-P/HC) was chosen as an inexpensive candidate for CH_4_ detection. Dynament also provides a dual gas NDIR sensor (MSH-DP/HC/CO_2_) designed to measure both CO_2_ and CH_4_ concentrations, which is attractive for applications requiring both low-cost and portability. Finally, CO_2_ and CH_4_ Gascard (sold by GHG Analytical) sensors were included, even though they were more expensive than the other chosen NDIR sensors. However, the Gascard sensors are still significantly less expensive than typical bench-top instruments. The inclusion of pressure and temperature sensors make them attractive enough to compensate for the expense.

The low-cost chemiresistive-based CH_4_ sensors studied here are typically used in gas warning systems [25]. Selected sensors include the MQ-4 from Hanwei Electronics and TGS-2600, TGS-2610, and TGS-2611 manufactured by Figaro Engineering Inc. sensors. The TGS sensors are used in commercial CH_4_ detectors, and the TGS-2600 sensor has been previously evaluated for atmospheric applications [26,27]. There are several different MQ versions optimized for hydrocarbon sensing. The MQ-4 sensor was chosen as this variant was specifically tuned for CH_4_. Chemiresistive sensors required a minimum conditioning period or “burn-in” time. This “burn-in” time is a period (often exceeding 24 h) during which the heating element is allowed to constantly heat the sensing element of the sensor. The “burn-in” time was met or exceeded for all chemiresistive sensors.

### 2.2. Analyte Preparation and Sensor Testing

**Figure 1:**
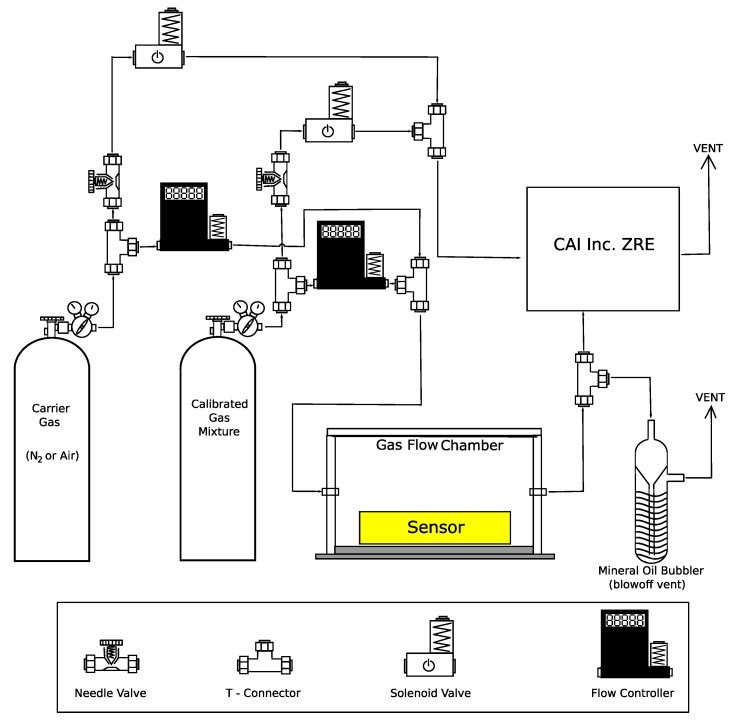
Component diagram of controlled gas exposure apparatus with chamber for diffusion-type sensors.

To test these sensors under controlled conditions, a gas mixing apparatus (Figure 1) was constructed which prepares gas mixtures of known concentration by controlling the positive pressure flow of gas streams into a mixing chamber. A high-quality bench-top analyzer (California Analytical Instruments, Inc. ZRE Non-Dispersive Infrared Analyzer), sensitive to both CO_2_ and CH_4_, was used as a precision reference. This setup allows gas flows of a known concentration to be prepared from a calibrated gas cylinder by mixing with a carrier gas. The calibrated gas contained a mixture of gases at concentrations listed in Table 1 depending on the specific experiment being performed. Since the objective is to address the accuracy, precision, sensitivity, and limits of detection, only CO_2_ and CH_4_ were utilized. However, selectivity requirements must be considered when these sensors are integrated into a monitoring platform. For CO_2_ experiments, the carrier gas was typically nitrogen. For CH_4_, medical grade air was utilized as a carrier gas since the chemiresistive sensors required oxygen to correctly measure the CH_4_ concentration. All reported concentrations were determined using the bench-top analyzer. Thus, the concentration ranges and all related values are reported here not as whole numbers but direct observations.

The calibrated gas mixtures were provided by and certified within ±2% by Airgas Inc. The calibrated gas was diluted using either air or nitrogen gas by a set of mass flow controllers to produce specific concentration of the analyte gases. The solenoid valves in the gas mixing apparatus were used to send either the undiluted calibrated gas or the carrier gas to analyzer for calibration purposes. This ability allows the analyzer to be periodically calibrated. The uncertainty in the gas concentration results in a systematic error. Around atmospheric levels of CH_4_, this error is less than 0.4 ppm. For atmospheric levels of CO_2_, the error is approximately 8 ppm. Since the fluctuations around the mean were independent of the exact concentration (see Section 3.3), this systematic error will not effect the results from the baseline noise or the International Union of Applied Chemistry (IUPAC) limit of detection, discussed below. For the sensor calibrations, uncertainty in the absolute concentration of the carrier gas will introduce an error in the intercept but not the slope. While any systematic error will have small effect on the calibration-corrected limit of detection, the random uncertainty dominates any potential systematic bias of a few ppm.

**Figure 2:**
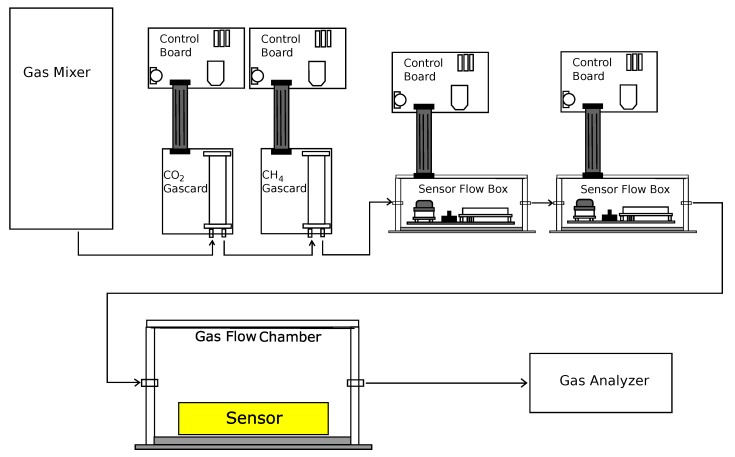
The diagram shows the flow of gas from the mixing apparatus to Gascard sensors, small enclosures containing sensors, the large gas flow chamber, and finally to the California Analytical Instruments, Inc. ZRE Non-Dispersive Infrared Analyzer. The gas mixing apparatus is shown in Figure 1. The reported concentrations are based on the value detected by the California Analytical Instruments, Inc. ZRE Non-Dispersive Infrared Analyzer.

Multiple sensors were operated concurrently as shown in Figure 2. The “Gas Mixer” in this diagram is the gas mixing apparatus shown in Figure 1, and the “Gas Analyzer” is the California Analytical Instruments, Inc. ZRE Non-Dispersive Infrared Analyzer. There are two types of sampling methods used by the selected sensors; diffusion and flow (see Tables S1 and S2 in the Supplementary Materials). Flow sampling sensors are designed such that the analyte gases must physically pass through a sampling chamber within the sensor. In terms of applications, these sensors typically require additional filters to be added upstream to prevent inclusion of particulate matter, which may damage the sensor or introduce measurement error. In the experimental setup (Figure 2), the flow capable sensors were connected directly to gas flow from the mixing apparatus. In addition to the flow sensors, another common sampling technique relies on diffusion through a fine mesh screen or film. The diffusion method allows the sensing element to be exposed to the analyte gas while still being protected from particulate contaminants. This method also eliminates the extra pump and particulate filters required for sensors that require gas flow. The small “sensor flow box” enclosures depicted in Figure 2 contained the K-30 CO_2_ sensor and a socketable chemiresistive CH_4_ sensor (such as the MQ-4 or TGS series sensors). The larger “gas flow chamber” contained the COZIR, Telaire, Dynament, and K-30 sensors. Given the large total volume, data collected from the sensors in this chamber were analyzed only after the system reached a constant concentration following the introduction of a gas with known concentration of CO_2_ or CH_4_. In some experiments, performed to accurately measure the response time for chemiresistive sensors, a smaller sensor enclosure (internal volume 2.54 cm^3^) was used to minimize the time to reach a stable concentration.

### 2.3. Sensor Output and Data Collection

(1) $$\varsigma =\frac{V_{measured}}{V_{cc}-V_{measured}}\times \frac{1}{R_{ref}}$$

The output of each sensor was logged as a function of time using software provided by the manufacturer as part of development kits when provided, or using an Arduino microcontroller with a prototyping board and microSD card. Due to the unique interface requirements of each sensor, development kits were purchased when possible. In all cases, the internal electronics within the selected optical sensors process the option data and provide a digital output. For example, the K-30 reports the concentration directly, while the Gascard reports a digital value between zero and one, with one being the maximum concentration (30,000 ppm CO_2_ for the sensor used in this study). For the Gascard, scaling was applied to the output before analyzing. The chemiresistive sensors were energized using the recommended voltage for the heater element and the response was measured using a 12-bit A/D converter, after buffering and filtering, across a reference resistor (a 10 kΩ resistor was used for $R_{ref}$). The A/D converter was referenced using the 5 V supply, which was also connected to the sensing element of the chemiresistive sensor. Equation (1) describes the conversion of the measured voltage ($V_{measured}$) to the conductivity (ς), measured in Siemens (S).

### 2.4. Calibration and Fitting Procedures

Calibration curve of each sensor was determined by varying concentrations and recording the output with time. The CO_2_ sensors were calibrated using gas concentrations from 34.5 to 1020 ppm. For the CH_4_ sensors, calibration curves were generated from gas concentrations between 1.85 and 995 ppm. The procedure starts with a constant flow of the carrier gas until stable baseline is obtained. Next, the analyte gas at a known concentration is introduced. After 24 h, the carrier gas is reintroduced and the system is again allowed to stabilize before the next measurement. During this procedure, concentration data from each sensor was continually collected. From these data, the average baseline and response to each gas concentration was extracted. The average response at each concentration was determined from the data collected after the system stabilized (include any overshoot or ringing) after each new concentration.

(2) $$f\left(x\right)=\frac{a\times b\times x}{1+b\times x}$$

The fitting procedures for the optical and chemiresistive sensors differ. The raw output of the optical absorption-based sensors is expected to follow Beer-Lambert law. In all cases, the internal electronics within the selected optical sensors process the absorbance and provide a digital output. For the optical sensors, the accuracy and precision of the internal calibration are quantified by linear regression. The calibration curves for the chemiresistive are non-linear, and modeled using a Langmuir-like or Langmuirian form (Equation (2)). Equation (2) converges to a linear function when the ppm approaches zero, which simplifies the limit of detection calculations discussed below. The parameters *a* and *b*, along with the asymptotic standard errors, are determined by fitting the experimental data using the Levenberg-Marquardt algorithm in gnuplot [28]. Additional non-linear functions have been suggested by kinetic analysis [29,30]. Of these, a power law model, where the log of the output is proportional to the log of the concentration in ppm, is considered.

### 2.5. Precision and Baseline Noise Tests

Precision of the sensors was determined by a 20 h to 30 h data collection period at a known concentration and uniform flow. These experiments were performed around the baseline atmospheric concentration for each analyte gas, which is approximately 400 ppm for CO_2_ [12,13] and under 2 ppm for CH_4_ [14,15,16]. For CO_2_, 400 ppm was utilized, and for CH_4_, 0 ppm of methane was used for the baseline noise measurements.

### 2.6. Limit of Detection Determination

The limit of detection is the minimum concentration that can be detected as significantly different from the background [31,32,33]. IUPAC defines the limit of detection as three times the standard deviation (σ) from the background. For the sensors discussed here, the raw output from the sensors must be transformed into a concentration, and any uncertainties in the calibration will affect the limit of detection. For measurements requiring a calibration curve, Long and Winefordner provide a review of the various definitions as well as several examples [31]. For optical sensors, their procedure to include the uncertainties in the slope and intercept in the calculated limit of detection was utilized. For the non-linear chemiresistive sensors, the asymptotic standard uncertainties in *a* and *b* were propagation to correct the IUPAC limit of detection.

## 3. Results and Discussion

### 3.1. Sensor Response

**Figure 3:**
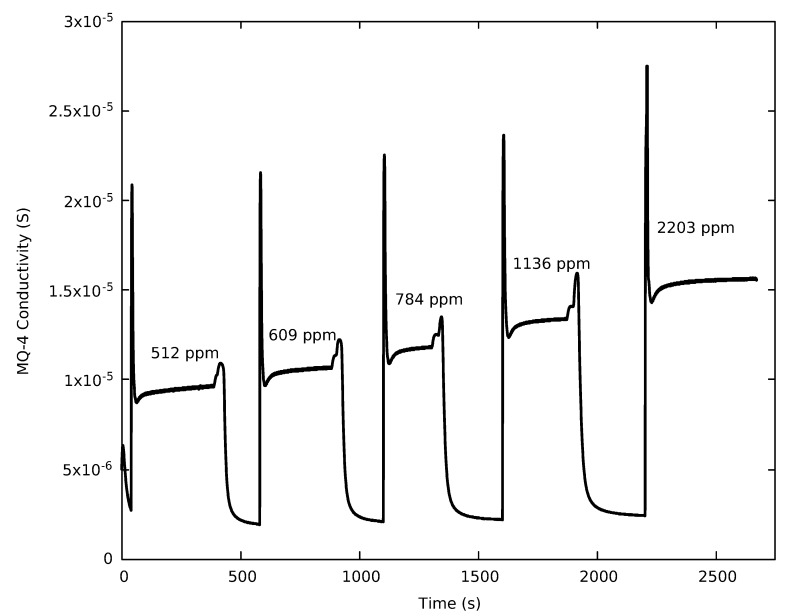
The MQ-4 response to difference CH_4_ concentrations determined by the California Analytical Instruments, Inc. ZRE Non-Dispersive Infrared Analyzer.

In general, all selected optical sensors responded quickly to an increase in CO_2_ inside the environmental chamber, with the response only limited by the internal sampling rate of the sensor. The chemiresistive sensors exhibit more complex behavior when exposed to a rapid change in concentration. Figure 3 depicts a plot of the response of an MQ-4 sensor upon exposure to five different concentrations of CH_4_ between 500 ppm and 2200 ppm, which could represent a CH_4_ leak. In our setup, the MQ-4 produces significant overshoot. Some of the overshoot is inherent to the sensor, but could also be a result of the small interruption in the gas flow during a concentration change. For all concentrations, a stable value within 2.5% of the mean was produced after 78 ± 10 s, when averaged over all experiments. The settling time for the MQ-4 sensor did not appear to be concentration dependent. Shorter settling time and less overshoot was observed for the TGS sensors.

### 3.2. Calibration Result

**Figure 4:**
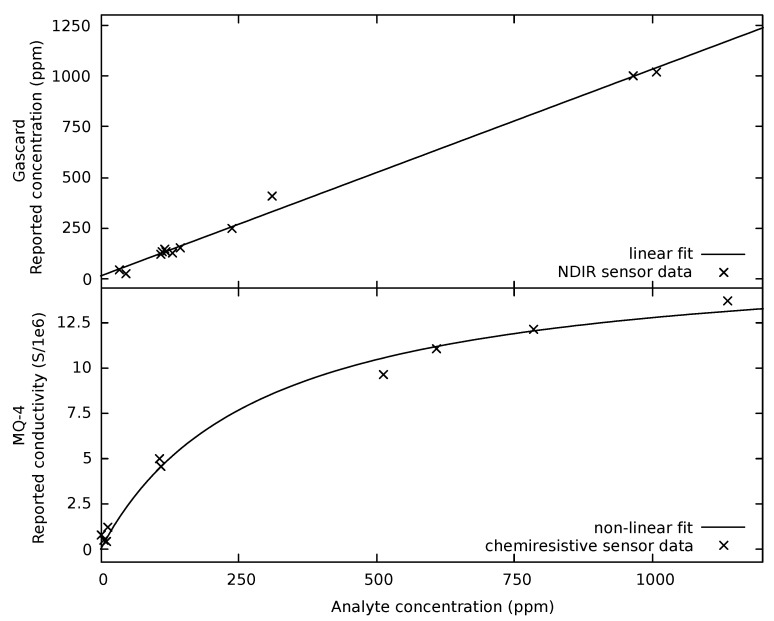
The upper panel of the figure shows the reported concentration from a Gascard optical CO_2_ sensor along with the expected linear fit. The lower panel of the figure shows reported conductivity measured from an MQ-4 chemiresistive CH_4_ sensor along with a fit to the expected non-linear fit. The analyte gas at various concentrations was generated by the gas mixing apparatus (Figure 1) and measured using California Analytical Instruments Inc. ZRE Non-Dispersive Infrared Analyzer.

Typical calibration plots for an optical and a chemiresistive sensors are shown in Figure 4. The analyte concentration (*x*-axis) was generated by the gas mixing apparatus (Figure 1) and measured using California Analytical Instruments Inc. ZRE Non-Dispersive Infrared Analyzer. The data points are the measured values at each concentration, while the line is a fit to the data. The upper plot in Figure 4 is the CO_2_ calibration curve obtained from the Gascard sensor, along with the linear fit. The *y*-axis is concentration reported by the sensor. The slope is 1.02±0.02 indicating an excellent calibration and precision. There is a significant gap between the highest two CO_2_ concentrations and the next highest, which could potentially bias the linear regression. However, elimination of these two highest points does not change the slope of the fit. Above their respective detection limits, good linear fits were obtained for each optical sensor. Linear regression results show the slopes for the selected sensors are all close to one, indicting good precision. For the CO_2_ sensors, the slopes range from 1.11 to 0.86, with the MSH-P/CO_2_ and Telair as the high and low outliers, respectively. Excluding these sensors, the average slope is 0.99. For the CH_4_ sensors, the slopes are 0.98 (Gascard), 1.03 (MSH-DP/HC/CO_2_), and 1.20 (MSH-P/HC). The fact that a majority of the slopes are close to one is expected as the optical path length and the physical properties of the analyte determine the response. In contrast to the slopes, the value of the vertical intercept or the response at zero concentration vary widely, exceeding several 100 ppm in many cases. Large variations are found even between sensors from the same manufacturer. Since the intercept depends on the intensity of the source and the detector, these variations are not unexpected. To address this issue, a majority of the optical sensors tested provide an easy way to adjust the intercept. For example, the K-30 sensors can auto-calibrate the zero based on known average background level of CO_2_. In general, it was found the intercept must be adjusted before use.

The lower plot in Figure 4 shows the measured conductivity (*y*-axis) of a MQ-4 chemiresistive CH_4_ sensor as a function of CH_4_ concentration. The solid line is a non-linear fit to a Langmuirian form (Equation (2)). The responses above 500 ppm were generated from the data in Figure 3. Additional points were generated at concentrations close to zero to better determine the Limit of Detection, which relies on the initial slope of the calibration curve close to zero. For ppm concentration above 500 ppm, acceptable results were also obtained using a power law model. However, this model produced a fit that increased too rapidly with ppm at lower concentration. At high concentrations, this model did not accurately represent a real sensor where the response approaches a constant. A lower concentration reference gas was required to precisely prepare an analyte gas at 100 ppm and below. This action resulted in a gap between 100 and 500 ppm in the lower plot. It is also significant that the chemiresistive sensors show a rapid increase in conductivity with concentration below 100 ppm when compared to the optical sensors.

Unlike optical sensors for CO_2_, which only needs reading at zero ppm to be experimentally established, chemiresistive sensors require the measurement of a complete non-linear calibration curve to ensure accuracy and precision. The output of the chemiresistive sensors are also dependent on temperature and humidity, further complicating the calibration [34]. Although the selected chemiresistive sensors have the requisite precision to provide a clear indication of a change (based on number of σ above the noise, see Table 2 along with the associated discussion below) at the ppm level, obtaining an accurate result is challenging.

### 3.3. Precision and Baseline Noise Tests

**Figure 5:**
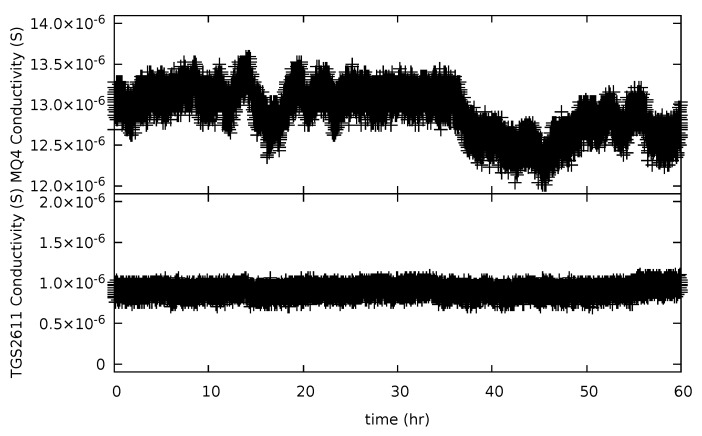
Long term fluctuations in the measured conductivity as a function of time are shown for the MQ-4 (upper panel) and TGS-2611 (lower panel) chemiresistive sensors. These baseline responses were collected at approximately 0 ppm CH_4_, representing the typical atmospheric concentrations of under 2 ppm [14,15,16]) using medical grade breathing air.

The measurement precision and baseline noise of each sensor was performed as described in Section 2.5. The precision of the sensors was determined by a 20 h to 30 h data collection time at a known concentration (400 ppm for CO_2_, which is the typical atmospheric concentration [12,13], and 0 ppm for CH_4_, representing the typical atmospheric concentrations of under 2 ppm [14,15,16]). In general, the optical sensors displayed flat response or baseline response with time Figure 5 shows a typical data set for the TGS-2611 and MQ-4 sensor. The TGS-2611 sensor displayed significantly less variation in conductivity and baseline drift than the MQ-4 sensor. In addition, chemiresistive sensors typically display baseline changes due to humidity and temperature, which must be taken into account in monitoring applications.

Since the initial Fourier analysis showed no significant periodic variations, distribution of the digitized sensor output around the mean response of the sensor was utilized to quantify the precision and baseline noise. The data stream (sensor output with time) from each sensor was subtracted from the mean response of the sensor, and a histogram of these differences was created. Since the digitized sensor outputs have a finite number of possible output values, no additional bins were created while producing the analysis. Although the data utilized for this analysis were obtained at typical environmental concentrations, additional experiments found that the measured deviation around the mean for each sensor was independent of the concentration of analyte gas. There was insignificant correlation between the concentration of the analyte gas and the σ obtained at each concentration, as quantified by the Pearson’s Correlation Coefficient (ρ=−0.173).

**Figure 6:**
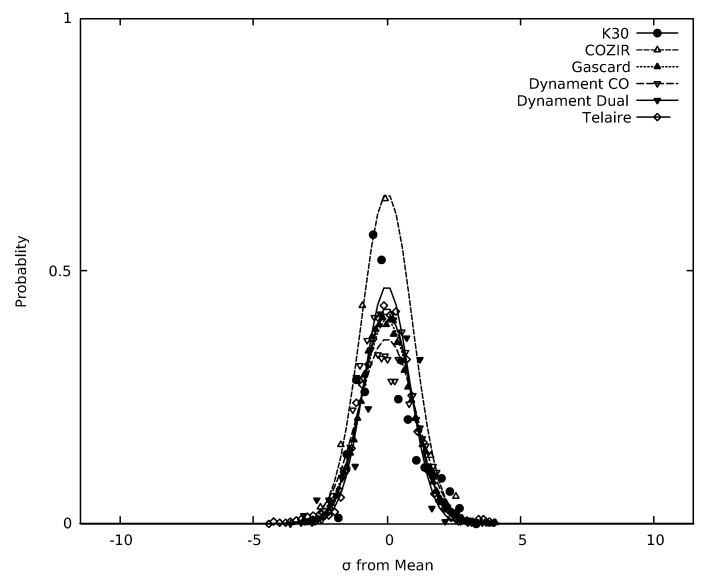
Frequency distribution of the digitized sensor output around the mean response of the sensor (points), along with Gaussian non-linear for each CO_2_ sensor around the typical environmental baseline concentration of 400 ppm. Any deviation between the center of the Gaussian fit and the mean are due to significant asymmetry of the frequency distribution around the mean.

**Figure 7:**
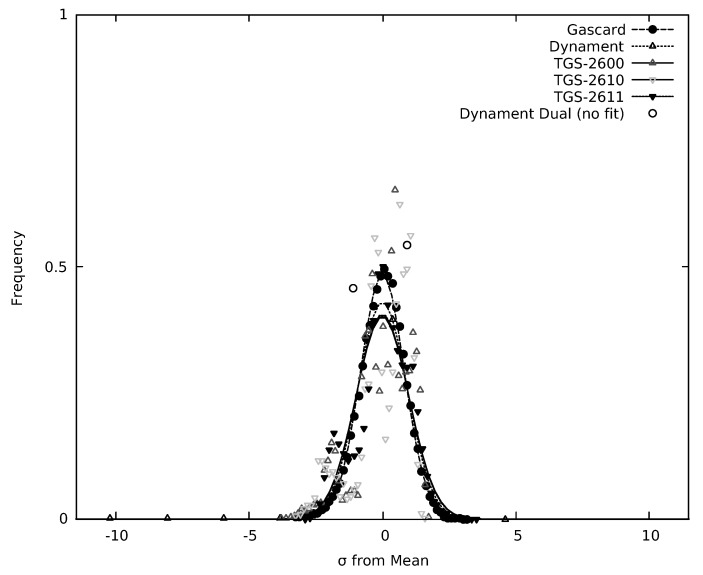
Frequency distribution of the digitized sensor output around the mean response of the sensor (points), along with Gaussian non-linear fit for each CH_4_ sensor around the typical environmental baseline concentration of 0 ppm. Any deviation between the center of the Gaussian fit and the mean are due to significant asymmetry of the frequency distribution around the mean.

The resulting histograms, along with a best-fit Gaussian peak, are shown in Figure 6 and Figure 7 for the CO_2_ and CH_4_ sensors, respectively. In Table 2, $\sigma_{GAUSS}$ is the standard deviation determined from the Gaussian fit. The standard deviation (σ) calculated from the background subtracted sensor data is almost identical to $\sigma_{GAUSS}$. In Figure 6 and Figure 7, the abscissa (*x*-coordinate) was scaled by the standard deviation of the data set and the area normalized to one. The resolution of the dual gas Dynament (MSH-DP/HC/CO_2_) sensor was insufficient to properly determine the fluctuations around the mean, as this sensor oscillated between two values with time at the given concentration. The limited responses are consistent with the resolution stated in their documentation, which lists 0.01% or 100 ppm as the low end of the concentration range. The fluctuation around the mean for the single gas Dynament (MSH-P/CO_2_) sensor is slightly less that the stated resolution of 50 ppm.

To determine the quality of the Gaussian fits in Figure 6 and Figure 7, the root-mean-squared error (RMSE) between the fit and the experimentally generated histogram was calculated for each sensor. These values are also listed in Table 2. For RMSE, a lower value indicates that the Gaussian function fits closely to the data points, whereas a higher value indicates a poorer fit. The values listed in Table 2 range from roughly 0.150 to 0.300. As the probability density function to which the data were fit was normalized to 1, the RMSE values are unitless. Since RMSE is a measure of fit, these values can be used in conjunction with $\sigma_{GAUSS}$ to characterize the sensors.

Of the CO_2_ sensors (see Figure 6 and Table 2), the Gascard and K-30 sensors produced the smallest standard deviation around the mean, $\sigma_{GAUSS}$, or the highest precision. The $\sigma_{GAUSS}$ value of the Telaire sensor was approximately two times that of either the Gascard or the K-30 sensor. The COZIR and Dynament (MSH-P/CO_2_) sensors generated $\sigma_{GAUSS}$ values which were significantly greater than that of the Gascard, Telaire and K-30 sensors. Finally, Table 2) shows that the dual gas Dynament (MSH-P/HC/CO_2_) sensor produced the largest $\sigma_{GAUSS}$ or deviation around the mean. The fluctuations around the mean for Dynament sensors are still lower than the quoted resolutions in the manufacturer’s documentation of 50 and 100 ppm, respectively, for these two sensors.

The Gascard sensor for CO_2_ produced a normal response around the mean detected value with low RMSE. At times, there were some large fluctuations, several standard deviations around the mean response, in the output of the Gascard. This suggests that some minimal digital filtering may be required. For the K-30 sensor, a good Gaussian fit was produced (small RMSE). However, the distribution for the K-30 was skewed toward higher concentration values and appeared to contain two overlapping peaks. This caused the $\sigma_{GAUSS}$ to be slightly larger and the RMSE to be artificially inflated. Careful analysis of this double peak showed each peak had a similar standard deviation (σ), suggesting a small shift in the mean measured value. Since the K-30 sensor periodically adjusts for changing backgrounds in the firmware to ensure a normal output concentration of 400 ppm, the observed change in the reported mean concentration in the middle of the run was likely caused by this period background adjustment. It is also possible the periodic temperature and pressure variations, which were corrected by the Gascard sensor, were responsible for the observed skew in the K-30. In general, precise work requires incorporation of pressure and temperature sensors into potential environmental sensing units to allow ppm corrections to be performed at time of measurement.

The probability distribution of responses around the mean by the GE Telaire sensor is a single peak with a $\sigma_{GAUSS}$ that is approximately two to three times as large as that of the K-30 and Gascard sensors. Since the GE Telaire and K-30 sensor share comparable sensing mechanisms and path lengths, this larger $\sigma_{GAUSS}$ was initially surprising. A direct comparison of the response for both the Telaire and K-30 sensors both with and without ambient light, showed that, unlike the K-30 sensor, the Telaire sensor was sensitive to ambient light level. Given that long data collection times are required to produce reliable histograms, the ambient light conditions changed over the course of data collection. It was suspected that the larger deviations around the mean for the Telaire sensor and resulting $\sigma_{GAUSS}$ values are due to changes in the ambient light. Further experiments with the sensor shielded from ambient light substantiate this suspicion.

For the optical CH_4_ sensors (see Figure 7 and Table 2), the Gascard for CH_4_ produced a $\sigma_{GAUSS}$ with a low RMSE. The single gas Dynament (MSH-P/HC/) hydrocarbon sensor produced a very low $\sigma_{GAUSS}$ and performed well in terms of precision. The $\sigma_{GAUSS}$ for the dual-gas Dynament (MSH-DP/HC/CO_2_) sensor was not included in this table. As mentioned previously, the dual-gas Dynament (MSH-DP/HC/CO_2_) sensor only reported two values for CH_4_ around the mean rather than a distribution of several values. This result was consistent with the 100 ppm resolution quoted in the manufacturer’s documentation for concentrations of less than 10% CH_4_.

The result for the chemiresistive CH_4_ sensors are also shown in Figure 7, with a $\sigma_{GAUSS}$ and an RMSE result in Table 2. The distribution curve for the MQ-4 sensor was not included. Since the MQ-4 sensor displayed significant baseline drift when compared to the TGS-2611 sensor (Figure 5), the standard deviation was instead calculated directly from a relatively flat region of the baseline. This different treatment was not inconsistent with the use of the sensor in many applications where drift is a result of temperature and humidity. In general, the use of a dynamic background subtraction algorithm is required for chemiresistive sensors. The $\sigma_{GAUSS}$ results of the TGS-2600 and TGS-2610 were similar, as expected due to their comparable sensing mechanisms. The baseline noise, as quantified by $\sigma_{GAUSS}$, for the CH_4_ optimized TGS-2611 sensor was lower than the other TGS sensors.

### 3.4. Limits of Detection

The limits of detection were determined as discussed in Section 2.6 and are listed in Table 2. It should be noted these tests were carried out in a controlled environment and are best-case values. Of the tested CO_2_ sensors, the Gascard, K-30, and Telaire sensors have comparable low limits of detection. The COZIR and Dynament single analyte sensor (MSH-P/CO_2_) have the next highest limits of detection, and the Dynament dual analyte sensor (MSH-DP/HC/CO_2_) has the highest. These results reflect the ordering of $\sigma_{GAUSS}$ in Table 2. These results can also be correlated with the optical path lengths of the sensors—larger path lengths result in greater sensitivity.

The limit of detection for the Dynament single analyte CH_4_ sensor (MSH-P/HC) demonstrates the important influence of calibration uncertainties on the reported limit. For this sensor, the 3.54 ppm precision ($\sigma_{GAUSS}$) or deviation around the mean was significantly lower than the 50 ppm resolution quoted in the manufacturer’s documentation at the low-end of the 1% concentration range of the sensor. The IUPAC limit of detection is 10.6 ppm. After correcting for the calibration error, the limit of detection is 170 ppm. This larger limit of detection is more consistent with expectations based on the stated resolution of this sensor.

Of the tested chemiresistive CH_4_ sensors, the TGS-2611 sensor had the lowest limit of detection under controlled conditions, primarily due to its stable baseline, which influences both the background noise and the quality of the calibration. Similar sensitivities have been reported for the TGS-2600 [26]. Both the non-methane optimized TGS-2600 and TGS-2610 sensors had similar limits of detection which were slightly larger than the CH_4_ optimized TGS-2611 and the MQ-4. The MQ-4 sensor has precision between the TGS-2611 and both the TGS-2600 and TGS-2610 sensors. The 82 ppm limit of detection for the MQ-4 sensor is consistent with scatter in the calibration curve at concentration under 100 ppm (see Figure 3).

## 4. Conclusions

The performance of a sensor platform for environmental or industrial monitoring is sensitive to the cost and performance of the individual sensor elements. The detection limits, accuracy, and precision for a range of potential sensors for measuring ppm concentrations of CO_2_ and CH_4_ for low-cost monitoring instrumentation quantified under consistent conditions and experimental methodologies. These parameters were quantified around the baseline atmospheric concentration for each analyte gas, which is approximately 400 ppm for CO_2_ [12,13] and under 2 ppm for CH_4_ [14,15,16]. The selected sensors were also evaluated at gas concentrations exceeding 1000 ppm, in order to mimic a leak from a CO_2_ sequestration location or a gas well.

For CO_2_, the Gascard sensor had high-performance based on the detection limits and precision reported in Table 2, but is comparatively more expensive than the other sensors investigated. The Gascard sensor is also limited by reliance on active sampling, which requires a pump that introduces a source of additional power consumption, a mechanical failure point, and sampling complexity. Nevertheless, given that concentrations are typically reported in parts-per notation, the inclusion of temperature and pressure corrections on the Gascard sensor allow it to automatically correct the reported ppm value and eliminates the need for additional sensors. The lower cost K-30 and Telaire sensors also had good performance. The ability of the K-30 and Telaire sensors to operate by passive diffusion rather than mechanically pumped flow also reduces the cost and complexity of a potential monitoring system. The K-30 sensor, along with suggested corrections for improved accuracy, was also investigated for ambient air monitoring [35]. One unique feature of the K-30 sensor is that it has an option to autozero and correct reported output by assuming the minimum concentration of a multi-day run is 400 ppm. If implemented, this feature has advantages for remote instrumentation.

For low-cost chemiresistive CH_4_ sensors, the performance as expected at concentration greater than the measured limit of detection (Table 2) or, equivalently, greater than approximately 100 ppm. However, these sensors do not meet the ppm or even sub-ppm sensitivity required for environmental monitoring. The more expansive but still relatively low-cost optical NDIR CH_4_ sensors (the GasCard CH_4_ and MSH-P/HC) also preform well at higher concentrations, but lack the required sensitivity for monitoring environmental levels of CH_4_ around the global average. Although progress has been made to produce viable sensors [36], larger, more costly devices are still required for precision measurements at the ppm level. With an increasing focus on remote sensing technology, a new generation of higher-sensitivity low-cost CH_4_ sensors will be required to produce accurate data at expected atmospheric concentrations. The reported detection limits and precision for a range of available low-cost sensors, all measured using the same methodology and experimental equipment, provides a solid reference to which future improvements can be benchmarked against.

## Figures and Tables

**Table 1:** Calibrated gas mixtures used to prepare various gas mixtures with a carrier gas.

Carbon Dioxide	Methane	Balance Gas
3000 ppm	3000 ppm	nitrogen
100 ppm	100 ppm	nitrogen
0 ppm	20 ppm	nitrogen

**Table 2:** The first two numbered are the computed standard deviation in ppm of Gaussian fitted probability distributions and root-mean-squared error in the fit. The next two numbers are the computed International Union of Applied Chemistry (IUPAC) ($3\sigma_{GAUSS}$) and calibration uncertainty corrected Limits of Detections in ppm.

	Sensor	$\sigma_{\mathrm{GAUSS}}$ (ppm)	RMSE	IUPAC	Corrected ${}^{†}$
Carbon Dioxide	K-30 SE-0018	1.91	0.219	5.7	27.1
	COZIR AMB GC-020	14.1	0.304	42.3	80.6
	Gascard CO_2_	2.12	0.223	6.4	32.1
	MSH-DP/HC/CO_2_	86.4	0.197	260	254
	MSH-P/CO_2_	17.6	0.217	52.8	68.0
	Telaire T6615	4.42	0.185	13.3	31.1
CH_4_/Hydrocarbon	MQ-4	26.7 ${}^{‡}$		80.0	82.0
	Gascard CH_4_	35.7	0.222	110	151
	MSH-P/HC	3.54	0.152	10.6	170
	TGS-2600	36.1	0.225	110	117
	TGS-2610	37.1	0.237	111	113
	TGS-2611	5.4	0.208	16.3	16.3

${}^{†}$ A linear calibration was utilized for the optical sensor, while the chemiresistive utilized the non-linear Langmuirian fit. See text. ${}^{‡}$ This value was determined directly from the experimental response.

## Supplementary Materials

The following are available online at https://www.mdpi.com/1424-8220/19/14/3157/s1, Table S1: Manufacturer listed properties of evaluated CO_2_ sensors, Table S2: Manufacturer listed properties of evaluated CH_4_ sensors.

## Abbreviations

The following abbreviations are used in this manuscript:

CO_2_	Carbon dioxide
CH_4_	Methane
NDIR	Nondispersive Infrared
RMSE	Root-mean-squared error
