# Supplementary material for: Precision and Limits of Detection for Selected Commercially Available, Low-Cost Carbon Dioxide and Methane Gas Sensors
Source: Sensors (Basel). 2019 Jul 18;19(14):3157. doi: 10.3390/s19143157 (PMC6679274; doi:10.3390/s19143157)
Supplement: Supplementary file 1 [file sensors-19-03157-s001.pdf]

Supplementary Materials: Background and Properties of Selected Commercially Available, Low-cost Carbon Dioxide and Methane Gas Concentration Sensors

Wesley T. Honeycutt [1,†], M. Tyler Ley [2], and Nicholas F. Materer [3,*]

Selection Rational

The selection rational, sensing method, and selected properties obtained from the manufacturer documentation is tabulated below. Sensors can be generally categorized by detection method, which as optical absorption, chemiresistive (based on the resistance changes of a material due to chemical reaction with an analyte [1]), and electrochemical. Since studies have cited concerns with electrochemical gas concentration sensors, such as a short lifetime and lack of robustness [2], only optical and chemiresistive sensors were selected. The potential application of the selected sensors is also impotent in the selection. For this study, the application includes, but is not limited to, environmental monitoring of local gas concentration and detection of leaks around industrial locations. For such applications, the collection of multiple samples at several locations in a given area (possibility including places without power availability) is critical to obtain reliable results. Thus, with only two exceptions, sensors selected were all commercially available in large volumes (at least 1000 units) at low-cost (defined here as less than $100 per unit in bulk) The sensors were further selected based on the reported sensitivity at environmental concentrations of CO_2 (around 400 ppm [3,4]) and CH_4 (under 2 ppm [5–7]), and at concentrations of several thousand ppm, which simulate a potential leak. In the selection process, the cost, limit of detection, precision, accuracy, reliability, and power consumption are all important parameters, many of which are not reported the manufacture or cannot be directly compared to other similar sensors.

Sensing Technology

Lower cost optical sensors typically utilize nondispersive infrared (NDIR) sensing. This method utilizes a broad spectrum light source which is restricted by a narrow band pass filter across the absorbance maximum before reaching the detector. Since these sensors utilize the Beer-Lambert law to relate absorption to concentration, the calibration is only dependent on the geometry of the sensor and physical properties of the gas [8]. In general, NDIR detection is utilized for CO_2 due to its relatively large molar absorption coefficient, allowing for short path lengths to be used in devices. For CH_4, NDIR detection is limited due to its lower absorption coefficient and overlapping symmetric C-H stretches. The overlapping stretches makes CH_4 difficult to distinguish from other common aliphatic gases such as ethane and propane [9]. The selected lower cost chemiresistive sensors typicality detect CH_4 using a thin oxide film [2] and work by measuring resistance changes due to differences in the electron transport through the metal oxide film, in the presence of oxygen and target gases [10]. The resistance change is typically non-linear with the analyte concentration. The chemiresistive sensors are known to respond to a range of hydrocarbon gases [11], which should be considered when integrating these sensors into a sensor platform.

Selected Sensors

Table S1 lists the selected CO_2 sensors with important properties obtained from the manufacturer. Table S2 lists the CH_4 or hydrocarbon sensors and respective properties. The K-30, COZIR, Dynament, and Telaire sensors are all NDIR sensors. These sensors were chosen as low-cost, lightweight sensors with satisfactory detection parameters of CO_2. Dynament also provides a dual gas NDIR sensor (MSH-DP/HC/CO2/) designed to measure both CO_2 and CH_4 concentrations. This ability was attractive given low-cost and portability requirements. The CO_2 and CH_4 Gascard sensors sold by GHG Analytical were an order of magnitude more expensive than the other chosen NDIR sensors,

which have a cost between that of the lowest cost sensors on our list and that of the bench-top analyzers. Their specifications combined with the included pressure and temperatures compensation make them attractive enough to make up for the expense. In addition to the Gascard sensor, the Dynament hydrocarbon sensors (MSH-P/HC and MSH-DP/HC/CO2/) were chosen as inexpensive candidates for CH_4 detection. Chemoresistive sensors include the MQ-4 from Hanwei Electronics and TGS-2600, TGS-2610, and TGS-2611 manufactured by Figaro Engineering Inc. sensors. The TGS sensors are used in commercial CH_4 and air quality detectors. There are several different MQ versions optimized for hydrocarbon sensing. The MQ-4 sensor was chosen as this variant was specifically tuned for CH_4.

Table S1. Manufacturer listed properties of evaluated CO_2 sensors

Sensor	Supplier	Type	Sampling Method	Cal. Range	Op. Range
K-30 SE-0018	CO_2Meter	NDIR	flow or diffusion	0-5000 ppm	0-10000 ppm
COZIR AMB GC-020	CO_2Meter	NDIR	flow or diffusion	0-5000 ppm	0-10000 ppm
Gascard CO_2	GHG Analytical	NDIR	flow	0-50000 ppm	0-50000 ppm
MSH-P/CO2/NC/5/V/P/F	Dynament	NDIR	diffusion	0-2491 ppm	0-5000 ppm
MSH-DP/HC/CO2/NC/P/F	Dynament	NDIR	diffusion	100-2500 ppm	0-5000 ppm
Telaire T6615	General Electric	NDIR	flow or diffusion	0-2000 ppm	0-2000 ppm

Sensor	Warm Up	T	Humidity	Auto-cal	V Input	Avg. I
K-30 SE-0018	<1 min	0-50°C	0-95%	Yes	4.5-14 VDC	40 mA
COZIR AMB GC-020	<3 s	0-50°C	0-95%	Yes	3.25-5.5 VDC	1.5 mA
Gascard CO_2	30 s	0-45°C	0-95%	Yes	7-30 VDC	250 mA
MSH-P/CO2/NC/5/V/P/F	45 s	-20-50°C	0-95%	No	3.0-5.0 VDC	75-85 mA
MSH-DP/HC/CO2/NC/P/F	45 s	-20-50°C	0-95%	No	3.0-5.0 VDC	75-85 mA
Telaire T6615	10 min	0-50°C	0-95%	Yes	0-5 VDC	33 mA

Table S2. Manufacturer listed properties of evaluated CH_4 sensors

Sensor	Supplier	Type	Sampling Method	Cal. Range	Op. Range
MQ-4	Futurelec	chemiresistive	diffusion		200-10000 ppm
Gascard CH_4	GHG Analytical	NDIR	flow	0-50000 ppm	0-50000 ppm
MSH-P/HC/NC/5/V/P/F	Dynament	NDIR	diffusion	0-5000 ppm	0-10000 ppm
MSH-DP/HC/CO2/NC/P/F	Dynament	NDIR	diffusion	5000-11000 ppm	0-10000 ppm
TGS-2600	Figaro Engineering	chemiresistive	diffusion		1-30 ppm
TGS-2610	Figaro Engineering	chemiresistive	diffusion		1000-25000 ppm
TGS-2611	Figaro Engineering	chemiresistive	diffusion		500-10000 ppm

Sensor	Warm Up	T	Humidity	Auto-cal	V Input	Avg. I
MQ-4				No	5 VDC	<150 mA
Gascard CH_4	30 s	0-45°C	0-95%	Yes	7-30 VDC	250 mA
MSH-P/HC/NC/5/V/P/F	30 s	-20-50°C	0-95%	No	3.0-5.0 VDC	75-85 mA
MSH-DP/HC/CO2/NC/P/F	30 s	-20-50°C	0-95%	No	3.0-5.0 VDC	75-85 mA
TGS-2600				No	5.0±0.2 VDC	4.2±4 mA
TGS-2610				No	5.0±0.2 VDC	5.6±5 mA
TGS-2611				No	5.0±0.2 VDC	5.6±5 mA

Sensors with no listed warm-up time required 7-day burn-in time

References

1. Wetchakun, K.; Samerjai, T.; Tamaekong, N.; Liewhiran, C.; Siriwong, C.; Kruefu, V.; Wisitsoraat, A.; Tuantranont, A.; Phanichphant, S. Semiconducting Metal Oxides as Sensors for Environmentally Hazardous Gases. *Sensors and Actuators B: Chemical* **2011**, *160*, 580 – 591. doi:https://doi.org/10.1016/j.snb.2011.08.032.
2. Neri, G. First Fifty Years of Chemoresistive Gas Sensors. *Chemosensors* **2015**, *3*, 1–20. doi:10.3390/chemosensors3010001.

3. Blasing, T. Recent Greenhouse Gas Concentrations. Technical report, U. S. Department of Energy, CDIAC, 2016.
4. Dlugokencky, E. Trends in Atmospheric Methane. Technical report, NOAA/ESRL, 2016.
5. Turner, A.J.; Jacob, D.J.; Benmergui, J.; Wofsy, S.C.; Maasakkers, J.D.; Butz, A.; Hasekamp, O.; Biraud, S.C. A Large Increase in U.S. Methane Emissions over the Past Decade Inferred from Satellite Data and Surface Observations. *Geophysical Research Letters* **2016**, *43*, 2218–2224. doi:10.1002/2016GL067987.
6. Bamberger, I.; Stieger, J.; Buchmann, N.; Eugster, W. Spatial Variability of Methane: Attributing Atmospheric Concentrations to Emissions. *Environmental Pollution* **2014**, *190*, 65–74. doi:10.1016/j.envpol.2014.03.028.
7. Dlugokencky, E.; Tans, P. Trends in Atmospheric Carbon Dioxide. Technical report, NOAA/ESRL, 2016.
8. Bacsik, Z.; Mink, J.; Keresztury, G. FTIR Spectroscopy of the Atmosphere. I. Principles and Methods. *Applied Spectroscopy Reviews* **2004**, *39*, 295–363. doi:10.1081/ASR-200030192.
9. Coblentz Society, Inc.. Evaluated Infrared Reference Spectra. In *NIST Chemistry WebBook*; Lindstrom, P.J.; Mallard, W.G., Eds.; Number 69 in NIST Standard Reference Database, National Institute of Standards and Technology: Gaithersburg MD, 20899.
10. Albert, K.J.; Lewis, N.S.; Schauer, C.L.; Sotzing, G.A.; Stitzel, S.E.; Vaid, T.P.; Walt, D.R. Cross-Reactive Chemical Sensor Arrays. *Chemical Reviews* **2000**, *100*, 2595–2626. doi:10.1021/cr980102w.
11. Sekhar, P.K.; Kysar, J.; Brosha, E.L.; Kreller, C.R. Development and Testing of an Electrochemical Methane Sensor. *Sensors and Actuators B: Chemical* **2016**, *228*, 162 – 167. doi:https://doi.org/10.1016/j.snb.2015.12.100.
